# *catena*-Poly[[[diaqua­manganese(II)]-bis­[μ-1,3-bis­(1*H*-imidazol-1-ylmeth­yl)benzene-κ^2^ *N* ^3^:*N* ^3′^]] dinitrate]

**DOI:** 10.1107/S1600536811039882

**Published:** 2011-10-05

**Authors:** Xiao-Dan Wang, Guang-Feng Hou, Ying-Hui Yu, Jin-Sheng Gao

**Affiliations:** a College of Chemistry and Materials Science, Heilongjiang University, Harbin 150080, People’s Republic of China; b Engineering Research Center of Pesticide of Heilongjiang University, Heilongjiang University, Harbin 150050, People’s Republic of China

## Abstract

In the title compound, {[Mn(C_14_H_14_N_4_)_2_(H_2_O)_2_](NO_3_)_2_}_*n*_, the Mn^II^ ion is located on an inversion center and is coordinated by four N atoms from four 1,3-bis­(1*H*-imidazol-1-ylmeth­yl)benzene (*L*) ligands and two water mol­ecules in a distorted octa­hedral geometry. Two *L* ligands are related by a centre of symmetry and bridge Mn^II^ ions, forming a positively charged polymeric chain in [101]. Uncoordinated nitrate anions further link these chains into layers parallel to the *ac* plane *via* O—H⋯O hydrogen bonds.

## Related literature

For details of the synthesis, see: Yang *et al.* (2006 ▶). For related structures, see: Dobrzańska *et al.* (2008 ▶); Dobrzańska (2009 ▶); Yao *et al.* (2008 ▶).
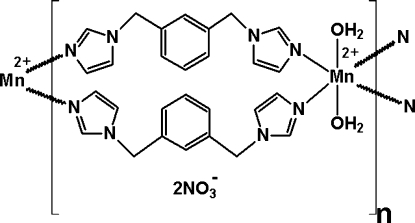


## Experimental

### 

#### Crystal data

[Mn(C_14_H_14_N_4_)_2_(H_2_O)_2_](NO_3_)_2_
*M* *_r_* = 691.58
Triclinic, 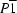

*a* = 8.393 (7) Å
*b* = 9.843 (7) Å
*c* = 10.634 (7) Å
α = 98.11 (3)°
β = 108.42 (3)°
γ = 98.77 (3)°
*V* = 806.8 (10) Å^3^
*Z* = 1
Mo *K*α radiation
μ = 0.47 mm^−1^
*T* = 293 K
0.38 × 0.22 × 0.17 mm

#### Data collection

Rigaku R-AXIS RAPID diffractometer
Absorption correction: multi-scan (*ABSCOR*; Higashi, 1995 ▶) *T*_min_ = 0.842, *T*_max_ = 0.923
6692 measured reflections
3567 independent reflections
2387 reflections with *I* > 2σ(*I*)
*R*_int_ = 0.031

#### Refinement

*R*[*F* ^2^ > 2σ(*F* ^2^)] = 0.049
*wR*(*F* ^2^) = 0.124
*S* = 1.07
3567 reflections
214 parameters
H-atom parameters constrained
Δρ_max_ = 0.23 e Å^−3^
Δρ_min_ = −0.31 e Å^−3^

### 

Data collection: *RAPID-AUTO* (Rigaku, 1998 ▶); cell refinement: *RAPID-AUTO*; data reduction: *CrystalClear* (Rigaku/MSC, 2002 ▶); program(s) used to solve structure: *SHELXS97* (Sheldrick, 2008 ▶); program(s) used to refine structure: *SHELXL97* (Sheldrick, 2008 ▶); molecular graphics: *SHELXTL* (Sheldrick, 2008 ▶); software used to prepare material for publication: *SHELXL97*.

## Supplementary Material

Crystal structure: contains datablock(s) global. DOI: 10.1107/S1600536811039882/cv5155sup1.cif

Additional supplementary materials:  crystallographic information; 3D view; checkCIF report

## Figures and Tables

**Table 1:** Hydrogen-bond geometry (Å, °)

*D*—H⋯*A*	*D*—H	H⋯*A*	*D*⋯*A*	*D*—H⋯*A*
O4—H41⋯O1	0.85	1.96	2.701 (3)	146
O4—H42⋯O3^i^	0.85	2.11	2.800 (3)	138

Symmetry code: (i) 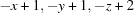.

**Fig. 1.**
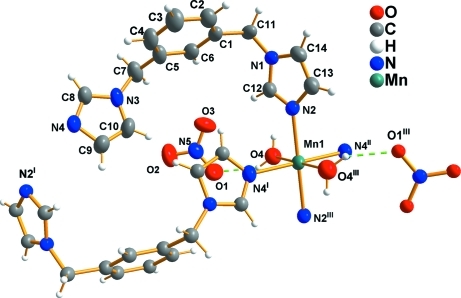
A portion of the crystal structure of (I) showing the atomic numbering and 50% probability displacement ellipsoids [symmetry codes: (i) 1-x, 1-y, 1-z; (ii) 1+x, y, 1+z; (iii) 2-x, 1-y, 2-z].

**Fig. 2.**
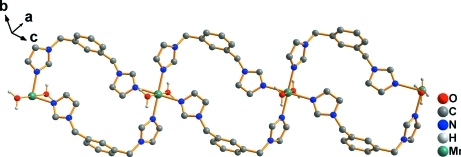
A portion of the positively charged polymeric chain in (I). C-bound H atoms omitted for clarity.

## supplementary crystallographic information

### Comment

In recent years, much study has been focused on using nitrogen-containing ligands to construct the supramolecular coordination compounds. The reason is that the supramolecular coordination assemblies not only own variety of architectures but also have the potential applications as functional materials. Recently, several supramolecular complexes based on the 1,3-bis(imidazol-1-yl-methyl)-benzene ligand (*L*) were reported (Dobrzanska *et al.*, 2008; Dobrzanska, 2009; Yao *et al.*, 2008). In this paper, we report the new title compound (I) synthesized by the reaction of 1,3-bis(imidazol-1-yl-methyl)benzene and manganese dinitrate in an aqueous solution, which forms an infinite one-dimensional chain structure.

In (I)  (Fig. 1), six-coordinated Mn^II^ ion locates on an inversion center. Its environment formed by four N atoms and two O atoms has a distorted octahedral geometry. Two ligands *L* related by centre of symmetry bridge Mn^II^ ions to form positively charged polymeric chain in [101] (Fig. 2). Uncoordinated nitrate anions link further these chains into layers parallel to *ac* plane *via* O—H···O hydrogen bonds (Table 1).

### Experimental

The 1,3-bis(imidazol-1-yl-methyl)benzene ligand was synthesized following the reference method (Yang *et al.*, 2006). 1,3-Bis(imidazol-1-yl-methyl)benzene (0.2143 g, 1 mmol) and 10 ml (0.1 mol/L) manganese dinitrate aqueous solution were dissolved in 10 ml ethanol. The mixture was stirred at 60 °C for 10 min. The resulting white precipitate was removed. Suitable single crystals were grown by slow evaporation from the mixed solution. White block crystals were obtained in 63 % yield based on manganese.

### Refinement

H atoms bound to C atoms were placed in calculated positions and treated as riding on their parent atoms, with C—H = 0.93 Å (aromatic); C—H = 0.97 Å (methylene), and with *U*_iso_(H) = 1.2*U*_eq_(C). Water H atoms were initially located in a differece Fourier map, but they were treated as riding on their parent atoms with O—H = 0.85 Å, and with *U*_iso_(H) = 1.5*U*_eq_(O). The abnormal reflections  (3 7 1), (3 -7 1), (-1 6 0), (-2 -6 1) and (1 5 0) have been omitted during the refinement.

### Crystal data

**Table:**

[Mn(C_14_H_14_N_4_)_2_(H_2_O)_2_](NO_3_)_2_	*Z* = 1
*M**_r_* = 691.58	*F*(000) = 359
Triclinic, *P*1	*D*_x_ = 1.423 Mg m^−^^3^
Hall symbol: -P 1	Mo *K*α radiation, λ = 0.71073 Å
*a* = 8.393 (7) Å	Cell parameters from 4631 reflections
*b* = 9.843 (7) Å	θ = 3.0–27.4°
*c* = 10.634 (7) Å	µ = 0.47 mm^−^^1^
α = 98.11 (3)°	*T* = 293 K
β = 108.42 (3)°	Block, colourless
γ = 98.77 (3)°	0.38 × 0.22 × 0.17 mm
*V* = 806.8 (10) Å^3^	

### Data collection

**Table:**

Rigaku R-AXIS RAPID diffractometer	3567 independent reflections
Radiation source: fine-focus sealed tube	2387 reflections with *I* > 2σ(*I*)
graphite	*R*_int_ = 0.031
ω scans	θ_max_ = 27.5°, θ_min_ = 3.0°
Absorption correction: multi-scan (*ABSCOR*; Higashi, 1995)	*h* = −10→10
*T*_min_ = 0.842, *T*_max_ = 0.923	*k* = −12→12
6692 measured reflections	*l* = −12→13

### Refinement

**Table:**

Refinement on *F*^2^	Primary atom site location: structure-invariant direct methods
Least-squares matrix: full	Secondary atom site location: difference Fourier map
*R*[*F*^2^ > 2σ(*F*^2^)] = 0.049	Hydrogen site location: inferred from neighbouring sites
*wR*(*F*^2^) = 0.124	H-atom parameters constrained
*S* = 1.07	*w* = 1/[σ^2^(*F*_o_^2^) + (0.0481*P*)^2^ + 0.2405*P*] where *P* = (*F*_o_^2^ + 2*F*_c_^2^)/3
3567 reflections	(Δ/σ)_max_ < 0.001
214 parameters	Δρ_max_ = 0.23 e Å^−^^3^
0 restraints	Δρ_min_ = −0.31 e Å^−^^3^

### Special details

**Table:**

Geometry. All esds (except the esd in the dihedral angle between two l.s. planes) are estimated using the full covariance matrix. The cell esds are taken into account individually in the estimation of esds in distances, angles and torsion angles; correlations between esds in cell parameters are only used when they are defined by crystal symmetry. An approximate (isotropic) treatment of cell esds is used for estimating esds involving l.s. planes.
Refinement. Refinement of F^2^ against ALL reflections. The weighted R-factor wR and goodness of fit S are based on F^2^, conventional R-factors R are based on F, with F set to zero for negative F^2^. The threshold expression of F^2^ > 2sigma(F^2^) is used only for calculating R-factors(gt) etc. and is not relevant to the choice of reflections for refinement. R-factors based on F^2^ are statistically about twice as large as those based on F, and R- factors based on ALL data will be even larger.

### Fractional atomic coordinates and isotropic or equivalent isotropic displacement parameters (Å^2^)

**Table:**

	*x*	*y*	*z*	*U*_iso_*/*U*_eq_	
O1	0.6014 (3)	0.7225 (3)	0.8857 (3)	0.0887 (8)	
C1	0.3199 (3)	0.0680 (3)	0.6811 (2)	0.0441 (6)	
C2	0.3261 (4)	−0.0279 (3)	0.5759 (3)	0.0540 (7)	
H2	0.3826	−0.1010	0.5939	0.065*	
C3	0.2480 (4)	−0.0154 (4)	0.4432 (3)	0.0663 (8)	
H3	0.2505	−0.0810	0.3722	0.080*	
C4	0.1670 (4)	0.0941 (3)	0.4168 (3)	0.0610 (8)	
H4	0.1166	0.1027	0.3276	0.073*	
C5	0.1594 (4)	0.1912 (3)	0.5202 (3)	0.0511 (7)	
C6	0.2370 (3)	0.1769 (3)	0.6523 (2)	0.0485 (6)	
H6	0.2331	0.2419	0.7232	0.058*	
C7	0.0640 (4)	0.3091 (4)	0.4925 (3)	0.0651 (9)	
H7A	−0.0582	0.2696	0.4525	0.078*	
H7B	0.0840	0.3715	0.5776	0.078*	
C8	0.0300 (4)	0.3810 (3)	0.2698 (3)	0.0557 (7)	
H8	−0.0755	0.3206	0.2225	0.067*	
C9	0.2586 (4)	0.5332 (4)	0.3180 (3)	0.0655 (8)	
H9	0.3438	0.6005	0.3103	0.079*	
C10	0.2641 (4)	0.4879 (4)	0.4333 (3)	0.0681 (9)	
H10	0.3512	0.5174	0.5173	0.082*	
C11	0.4068 (4)	0.0573 (3)	0.8265 (3)	0.0504 (6)	
H11A	0.3511	0.1026	0.8827	0.061*	
H11B	0.3933	−0.0409	0.8329	0.061*	
C12	0.6573 (4)	0.2604 (3)	0.9098 (3)	0.0478 (6)	
H12	0.5922	0.3292	0.9070	0.057*	
C13	0.8680 (4)	0.1583 (3)	0.9358 (3)	0.0524 (7)	
H13	0.9792	0.1431	0.9551	0.063*	
C14	0.7247 (4)	0.0567 (3)	0.8936 (3)	0.0511 (7)	
H14	0.7188	−0.0397	0.8784	0.061*	
N1	0.5897 (3)	0.1223 (2)	0.87739 (19)	0.0428 (5)	
N2	0.8254 (3)	0.2879 (2)	0.9461 (2)	0.0457 (5)	
N3	0.1169 (3)	0.3907 (3)	0.4016 (2)	0.0530 (6)	
N4	0.1113 (3)	0.4668 (2)	0.2149 (2)	0.0531 (6)	
N5	0.4591 (3)	0.6495 (3)	0.8143 (3)	0.0552 (6)	
O2	0.3562 (4)	0.6999 (3)	0.7364 (3)	0.1026 (9)	
O3	0.4241 (3)	0.5255 (2)	0.8242 (3)	0.0767 (7)	
O4	0.8056 (3)	0.5934 (2)	1.0595 (2)	0.0590 (5)	
H41	0.7729	0.6628	1.0273	0.089*	
H42	0.7398	0.5217	1.0653	0.089*	
Mn1	1.0000	0.5000	1.0000	0.04181 (18)	

### Atomic displacement parameters (Å^2^)

**Table:**

	*U*^11^	*U*^22^	*U*^33^	*U*^12^	*U*^13^	*U*^23^
O1	0.0743 (17)	0.0536 (14)	0.117 (2)	0.0082 (13)	0.0038 (16)	0.0225 (14)
C1	0.0396 (14)	0.0493 (15)	0.0368 (13)	−0.0001 (11)	0.0066 (11)	0.0134 (11)
C2	0.0550 (17)	0.0573 (18)	0.0498 (15)	0.0138 (14)	0.0155 (14)	0.0146 (13)
C3	0.079 (2)	0.076 (2)	0.0404 (15)	0.0232 (19)	0.0160 (15)	0.0071 (14)
C4	0.065 (2)	0.081 (2)	0.0344 (14)	0.0198 (17)	0.0108 (13)	0.0167 (14)
C5	0.0486 (16)	0.0669 (19)	0.0402 (13)	0.0150 (14)	0.0132 (12)	0.0205 (13)
C6	0.0516 (16)	0.0531 (16)	0.0367 (13)	0.0042 (13)	0.0131 (12)	0.0087 (11)
C7	0.066 (2)	0.088 (2)	0.0552 (17)	0.0299 (18)	0.0248 (16)	0.0338 (17)
C8	0.0487 (16)	0.0636 (19)	0.0472 (15)	0.0081 (14)	0.0037 (13)	0.0211 (13)
C9	0.0556 (19)	0.072 (2)	0.0518 (17)	−0.0038 (16)	0.0010 (14)	0.0159 (15)
C10	0.059 (2)	0.084 (2)	0.0426 (16)	0.0064 (18)	−0.0037 (14)	0.0134 (15)
C11	0.0515 (16)	0.0490 (16)	0.0394 (13)	−0.0061 (13)	0.0056 (12)	0.0140 (11)
C12	0.0471 (16)	0.0419 (15)	0.0471 (14)	0.0089 (12)	0.0047 (12)	0.0128 (11)
C13	0.0520 (17)	0.0491 (17)	0.0521 (15)	0.0151 (14)	0.0089 (13)	0.0140 (13)
C14	0.0639 (19)	0.0358 (14)	0.0477 (15)	0.0105 (13)	0.0101 (14)	0.0110 (11)
N1	0.0467 (12)	0.0379 (12)	0.0332 (10)	−0.0003 (10)	0.0026 (9)	0.0102 (8)
N2	0.0422 (13)	0.0405 (12)	0.0439 (12)	0.0038 (10)	0.0022 (10)	0.0098 (9)
N3	0.0522 (14)	0.0656 (16)	0.0412 (12)	0.0190 (12)	0.0084 (11)	0.0211 (11)
N4	0.0503 (14)	0.0571 (15)	0.0429 (12)	0.0083 (12)	0.0022 (10)	0.0172 (11)
N5	0.0592 (16)	0.0522 (15)	0.0580 (14)	0.0208 (13)	0.0195 (13)	0.0157 (12)
O2	0.098 (2)	0.101 (2)	0.0990 (19)	0.0415 (18)	0.0018 (16)	0.0411 (17)
O3	0.0809 (17)	0.0468 (14)	0.1141 (19)	0.0123 (12)	0.0465 (15)	0.0250 (12)
O4	0.0575 (12)	0.0531 (12)	0.0705 (13)	0.0177 (10)	0.0226 (11)	0.0174 (10)
Mn1	0.0392 (3)	0.0404 (3)	0.0387 (3)	0.0059 (2)	0.0039 (2)	0.0101 (2)

### Geometric parameters (Å, °)

**Table:**

O1—N5	1.237 (4)	C10—H10	0.9300
C1—C2	1.377 (4)	C11—N1	1.462 (4)
C1—C6	1.383 (4)	C11—H11A	0.9700
C1—C11	1.513 (3)	C11—H11B	0.9700
C2—C3	1.387 (4)	C12—N2	1.313 (3)
C2—H2	0.9300	C12—N1	1.340 (3)
C3—C4	1.375 (4)	C12—H12	0.9300
C3—H3	0.9300	C13—C14	1.347 (4)
C4—C5	1.376 (4)	C13—N2	1.376 (3)
C4—H4	0.9300	C13—H13	0.9300
C5—C6	1.387 (4)	C14—N1	1.364 (3)
C5—C7	1.518 (4)	C14—H14	0.9300
C6—H6	0.9300	N2—Mn1	2.243 (3)
C7—N3	1.469 (3)	N4—Mn1^i^	2.270 (2)
C7—H7A	0.9700	N5—O2	1.214 (3)
C7—H7B	0.9700	N5—O3	1.238 (3)
C8—N4	1.317 (4)	O4—Mn1	2.203 (2)
C8—N3	1.342 (3)	O4—H41	0.8499
C8—H8	0.9300	O4—H42	0.8501
C9—C10	1.352 (4)	Mn1—O4^ii^	2.203 (2)
C9—N4	1.361 (4)	Mn1—N2^ii^	2.243 (3)
C9—H9	0.9300	Mn1—N4^iii^	2.270 (2)
C10—N3	1.357 (4)	Mn1—N4^iv^	2.270 (2)
			
C2—C1—C6	119.1 (2)	N1—C12—H12	123.7
C2—C1—C11	120.7 (2)	C14—C13—N2	109.9 (3)
C6—C1—C11	120.2 (2)	C14—C13—H13	125.1
C1—C2—C3	120.0 (3)	N2—C13—H13	125.1
C1—C2—H2	120.0	C13—C14—N1	106.7 (2)
C3—C2—H2	120.0	C13—C14—H14	126.6
C4—C3—C2	120.0 (3)	N1—C14—H14	126.6
C4—C3—H3	120.0	C12—N1—C14	106.3 (2)
C2—C3—H3	120.0	C12—N1—C11	126.1 (2)
C3—C4—C5	121.0 (3)	C14—N1—C11	127.5 (2)
C3—C4—H4	119.5	C12—N2—C13	104.6 (2)
C5—C4—H4	119.5	C12—N2—Mn1	127.07 (18)
C4—C5—C6	118.4 (3)	C13—N2—Mn1	128.24 (19)
C4—C5—C7	121.6 (2)	C8—N3—C10	106.6 (2)
C6—C5—C7	120.0 (3)	C8—N3—C7	126.4 (3)
C1—C6—C5	121.5 (2)	C10—N3—C7	126.9 (2)
C1—C6—H6	119.2	C8—N4—C9	104.3 (2)
C5—C6—H6	119.2	C8—N4—Mn1^i^	124.1 (2)
N3—C7—C5	112.9 (2)	C9—N4—Mn1^i^	131.3 (2)
N3—C7—H7A	109.0	O2—N5—O1	119.9 (3)
C5—C7—H7A	109.0	O2—N5—O3	121.1 (3)
N3—C7—H7B	109.0	O1—N5—O3	119.0 (3)
C5—C7—H7B	109.0	Mn1—O4—H41	119.7
H7A—C7—H7B	107.8	Mn1—O4—H42	102.0
N4—C8—N3	112.3 (3)	H41—O4—H42	125.4
N4—C8—H8	123.8	O4^ii^—Mn1—O4	180.000 (1)
N3—C8—H8	123.8	O4^ii^—Mn1—N2	90.62 (9)
C10—C9—N4	110.8 (3)	O4—Mn1—N2	89.38 (9)
C10—C9—H9	124.6	O4^ii^—Mn1—N2^ii^	89.38 (9)
N4—C9—H9	124.6	O4—Mn1—N2^ii^	90.62 (9)
C9—C10—N3	106.0 (3)	N2—Mn1—N2^ii^	180.00 (11)
C9—C10—H10	127.0	O4^ii^—Mn1—N4^iii^	91.51 (9)
N3—C10—H10	127.0	O4—Mn1—N4^iii^	88.49 (9)
N1—C11—C1	112.2 (2)	N2—Mn1—N4^iii^	88.86 (9)
N1—C11—H11A	109.2	N2^ii^—Mn1—N4^iii^	91.14 (9)
C1—C11—H11A	109.2	O4^ii^—Mn1—N4^iv^	88.49 (9)
N1—C11—H11B	109.2	O4—Mn1—N4^iv^	91.51 (9)
C1—C11—H11B	109.2	N2—Mn1—N4^iv^	91.14 (9)
H11A—C11—H11B	107.9	N2^ii^—Mn1—N4^iv^	88.86 (9)
N2—C12—N1	112.6 (2)	N4^iii^—Mn1—N4^iv^	180.000 (1)
N2—C12—H12	123.7		

Symmetry codes: (i) *x*−1, *y*, *z*−1; (ii) −*x*+2, −*y*+1, −*z*+2; (iii) *x*+1, *y*, *z*+1; (iv) −*x*+1, −*y*+1, −*z*+1.

### Hydrogen-bond geometry (Å, °)

**Table:**

*D*—H···*A*	*D*—H	H···*A*	*D*···*A*	*D*—H···*A*
O4—H41···O1	0.85	1.96	2.701 (3)	146.
O4—H42···O3^v^	0.85	2.11	2.800 (3)	138.

Symmetry codes: (v) −*x*+1, −*y*+1, −*z*+2.
